# Racial and Ethnic Differences in Diabetes Care Quality in A National Sample of Cancer Survivors Relative to Non-Cancer Controls

**DOI:** 10.1007/s40615-024-02156-0

**Published:** 2024-09-04

**Authors:** Denalee M. O’Malley, Sarah Alavi, Jennifer Tsui, Cilgy M. Abraham, Pamela Ohman-Strickland

**Affiliations:** 1 Department of Family Medicine and Community Health, Rutgers Robert Wood Johnson Medical School, New Brunswick, NJ USA; 2 Rutgers Cancer Institute of New Jersey, New Brunswick, NJ USA; 3 Rutgers School of Public Health, Department of Epidemiology and Biostatistics, Piscataway, NJ USA; 4 Keck School of Medicine, University of Southern California, Los Angeles, CA USA; 5 Georgetown University Law Center, Georgetown University, Washington, DC USA

**Keywords:** Breast cancer, Colorectal cancer, Prostate cancer, Diabetes, Care disparities, Survivorship

## Abstract

**Background:**

Among cancer survivors, diabetes is associated with greater morbidity and mortality. The objective of this study is to describe racial/ethnic disparities in diabetes care quality (DCQ) among cancer survivors compared to non-cancer controls.

**Methods:**

We used Medical Expenditure Panel Survey Household Component data (2010–2018). Black, non-Hispanic White (NHW), and Hispanic respondents diagnosed with diabetes and cancer were frequency matched 1:5 to non-cancer controls. Multivariable logistic regression estimated associations for specific indices and overall DCQ by race/ethnicity stratified by cancer site/status in partially adjusted (not controlling for socioeconomic indicators) and fully adjusted models.

**Results:**

The final sample of 4775 included cancer survivors (*n* = 907 all cancers; *n* = 401 breast; *n* = 167 colon; *n* = 339 prostate) and non-cancer controls (*n* = 3868) matched by age, race/ethnicity, and year. In partially adjusted models, Black (adjusted odds ratio, AOR) 0.67 [95% CI 0.54–0.83]) and Hispanic (AOR 0.68 [95% CI 0.54–0.87]) non-cancer controls had significant disparities for overall DCQ compared to NHWs. Among cancer survivors, DCQ disparities for Black (AOR 0.62, [95% CI 0.4–0.96]) and Hispanics (AOR 0.60, [95% CI 0.38–0.97]) were identified. Among prostate cancer survivors, DCQ disparities were identified for Blacks (AOR 0.38; [95% CI 0.20–0.72]) and Hispanics (AOR 0.39; [95% CI 0.17–0.89]) compared to NHWs. Racial disparities among Black controls and Black prostate cancer survivors remained significant in fully adjusted models.

**Conclusion:**

Diabetes care disparities are evident among cancer survivors and salient among non-cancer controls. Strategies to promote health equity should target specific care indices among survivors and emphasize equitable DCQ strategies among Black and Hispanic communities.

## Introduction

In the United States (US), survivors of breast, colorectal (CRC), and prostate (PCa) cancers account for over half (~ 9 million) of the overall cancer survivor population [1]. These cancer sites are among the most prevalent in the US population, with over 600,000 additional cases diagnosed each year [2]. These cancer diagnoses are associated with individuals of older age, more treatable prognoses, and longer term survival. For patients over 40 years old, breast, colorectal, and prostate cancers are among the cancer sites with greatest risk of cardiovascular morality among cancer survivors [3]. Multi-morbidity, characterized by the presence of two or more co-occurring conditions, is common among aging populations and is a critical issue in cancer survivorship [4]. The cumulative effects of multimorbidity impacts health outcomes differently than individual diseases, and specific racial and ethnic groups are at higher risk for poor outcomes [5–8]. A recent study found that “complex cardiometabolic” clusters of multi-morbidity, which include both cancer and diabetes are associated with greater mortality risks than other multi-morbidity clusters [9]. Approximately one-fifth of the cancer survivor population also have a diagnosis of diabetes mellitus (DM) (20% breast, 20–24% CRC, and 18.9% prostate) [1, 10]. Among the US non-cancer adult population, crude prevalence data suggest 13% (34.1 million adults) of the general population have diabetes [11], and prevalence is disproportionately higher among racial and ethnic minority populations (e.g., non-Hispanic Blacks 18.4%, Hispanics 20.3%, and non-Hispanic Whites 11.9%) [12].

Diabetes diagnoses are associated with greater all-cause, cancer-, and diabetes-related morbidity and mortality among cancers survivors [10, 13]. Diabetes care quality is a health system factor of multi-morbidity management that may influence overall cancer and health outcomes for populations faced with managing cumulative social (e.g., racism, language barriers) and medical risks [12, 14–17]. Multiple factors contribute to inequities in cancer and diabetes prevalence and health outcomes. Social and structural issues known to influence cancer and diabetes risks include socioeconomic status (e.g., education, income, occupation), community and build environment (e.g., housing, environmental exposures), food environment (e.g., food deserts, food insecurity), health care (e.g., access, quality), and social context (e.g., support, racism, social capital) [18]. Several studies investigating diabetes care quality following a cancer diagnosis report that glucose control, medication adherence, and self-management behaviors decline after cancer diagnoses [19–23]. Recent care delivery research documented declines in diabetes care engagement the year after a cancer diagnosis, specifically declines in hemoglobin A1c (A1c) testing, low-density lipoprotein (LDL) testing, and receipt of all diabetes quality indicators [19, 24]. These studies focused primarily on cancer survivors receiving chemotherapy [20–23], had small sample sizes [22, 23], and did not investigate known racial and ethnic care delivery disparities in diabetes care quality.

To implement equitable care delivery strategies, the needs and structural barriers of medically and socially at-risk cancer survivors must be better understood. Additionally, the phase of care (e.g., prevention, survivorship) where targeting interventions has the greatest potential to optimize health outcomes must be identified. To address this, we conducted this retrospective study with the following objectives: (1) identify racial/ethnic disparities in the diabetes care delivery (i.e., A1c, LDL check, eye exam, flu shot, diet, receipt of diabetes education, office-based visits) among the breast, CRC, and PCa cancer survivor populations; and (2) compare diabetes care quality disparities among cancer survivors and non-cancer controls frequency matched by age, cohort year, and race.

## Methods

### Data Source and Study Population

We pooled data from the Medical Expenditures Panel Survey Household Component (MEPS-HC) from 2010 to 2018 to examine associations between quality of care and race/ethnicity by cancer type and history using IPUMS USA [25]. The MEPS-HC is an annual, nationally representative survey of the non-institutionalized US civilian population conducted by the Agency for Healthcare Quality and identified from a sub-sample of the National Health Interview Study (NHIS). MEPS-HC data assessed socio-demographics, health care conditions, and utilization [26]. Adult (i.e., ≥ 18 years old) respondents who self-reported a diabetes diagnosis formed the sampling frame of the study. Respondents with both type I and type II diabetes were included. We identified patients who had self-reported a diagnosis of diabetes and breast (*n* = 401), colorectal (*n* = 167), or PCa (*n* = 339) and frequency matched 1:5 by age (5-year intervals), race/ethnicity, and cohort year for patients with diabetes as non-cancer controls (*n* = 3868). Questions about previous diagnoses were framed with the following question stem, “Has a doctor or health professional ever told you that you have…” Responses from separate questions about previous diagnoses of diabetes or “sugar” diabetes, breast cancer, colon cancer, rectal cancer, and/or prostate cancer were used to identify the study sample.

Respondents were excluded if they did not have complete data for the diabetes care survey questionnaire, a self-reported questionnaire administered to patients with a reported diagnosis of diabetes. Additionally, respondents who responded “unknown” to any of the diabetes care survey questions were excluded. Respondents who reported a cancer history for any other type of cancer (e.g., types other than breast, colorectal, or PCa) and those who have been diagnosed with multiple cancers were excluded. For MEPS-HC, response rates ranged from 42.7 to 56.3% for the years of the study sample [27]. Additional information about MEPS-HC survey design and content are available elsewhere [28]. We used the IPUMS data platform to assemble the pooled data set. The Rutgers Institutional Review Board reviewed the study protocol and considered this study exempt research, since it involves only de-identified publicly available data.

### Variables of Interest

The dependent variables for this study were five diabetes care quality indicators based on clinical guidelines and the number of office-based visits in the past year. We based quality indicators on the self-reported responses of items on the Diabetes Care Survey about care received in the previous year, specifically: (1) A1c testing, (2) foot exams, (3) LDL testing, (4) dilated eye exams, and (5) flu shot. A1C test was coded as either guidelines met (2 +) or unmet (0–1) since clinical guidelines recommend tests every 6 months. All other indicators were also binary (service provided/not provided). For the composite score, we counted the number of services each participant received and then dichotomized that score into quality overall care versus inadequate care (4–5 versus 0–3 services). A final, separate indication of quality of care was the number of office visits for any type of provider for each patient over the year. Because these visits were not necessarily related to diabetes care, we kept this measure separate from the composite score.

We explored utilization disparities comparing survivors and non-cancer controls who had five or more annual office visits. The primary independent variable was race/ethnicity derived from responses to two questions that assessed race and ethnicity separately. Responses were collapsed and categorized: Black (inclusive of Hispanic ethnicity), non-Hispanic White (NHW), and Hispanic White. Only respondents that self-identified racially/ethnically as Black (inclusive of Hispanic ethnicity), non-Hispanic White, and Hispanic Whites were included. Respondents who did not report race or reported any other racial/ethnic identity (e.g., Asian, mixed race) were excluded from this analysis due to small samples in demographic categories.

All covariates used in these analyses were based on self-report. Age and gender sociodemographic variables were included. Age was retained in the complete data as a continuous covariate. Gender (male vs. female) was a binary variable. Socioeconomic (SES) variables included: education (≤ grade 12 vs. ≥ grade 13); insurance status (public/uninsured vs. private); income level (defined as a percentage of the poverty level and grouped into three categories: poor/low income (< 199% poverty line), middle income (200% to less than 400%,) and high income (≥ 400%)); and marital status (defined as: married, widowed and unmarried (e.g., divorced, separated, and never married)). Clinical variables included comorbidity covariates and current smoking status. Current smoker and comorbidity variables were coded into binary indicators based on positive responses to a series of questions structured, “have you ever been diagnosed with X comorbidity…” Cardiovascular diseases indicate respondents reported any of the following conditions: coronary heart disease, angina, myocardial infarction, stroke, or other related diseases.

### Analysis

Unweighted frequencies and estimated weighted percentages within each cancer type and non-cancer controls were summarized with socio-demographic, socioeconomic, and clinical characteristics. For each cancer type and non-cancer controls, we used Rao Scott chi-square to estimate weighted independent associations by race/ethnicity on overall diabetes care (based on composite score) and the receipt of each diabetes care services. Logistic regression models were used to estimate weighted unadjusted, partially adjusted, and fully adjusted odds ratio and 95% confidence intervals comparing Blacks and Hispanics versus NHWs for overall diabetes care and each diabetes indicator by cancer type and non-cancer controls. Fully adjusted models (presented in Table 4) controlled for demographic and clinical confounders which include socioeconomic status. We estimated “partially adjusted” models which did not adjust for socioeconomic indicators (e.g., poverty level, marital status, education, and insurance status) and presented both to provides insights about the racial and ethnic disparities that remain significant when SES factors are controlled. Because of matching, unadjusted ORs controlled for matching covariates where as adjusted odds rations (AORs) controlled for matching covariates as well as other specified covariates based on the model. Interactions between cancer status, cancer type, and race were tested via Wald chi-squares to determine whether cancer modified the effect of race on quality of care. All statistical analyses were performed using SAS (SAS Enterprise Guide 7.1). An alpha of 0.05 was used for significance for all analyses. All estimates were weighted using SAS survey command procedures to account for the MEPS complex survey design (i.e., adjusting weight, strata and cluster).

## Results

Table 1 shows respondent characteristics stratified by cancer site and status (e.g., breast, colorectal, PCas, non-cancer). The unweighted sample size was 4775 adults with diabetes who completed the diabetes care survey and self-reported race/ethnicity, which represents a weighted sample of 57,491,260 individuals with diabetes with 10,808,503 (14.8%) cancer survivors and 46,682,757 (81.2%) non-cancer controls.

**Table 1:** Participant characteristics by cancer site/status

	Breast (*n* = 401)		CRC (*n* = 167)		PCa (*n* = 339)		No-cancer (*n* = 3868)	
	Unweighted *n*	Weighted %	Unweighted *n*	Weighted %	Unweighted *n*	Weighted %	Unweighted *n*	Weighted %
Gender								
Male	NA		98	67.4	339	100.00	2152	46.1
Female	401	100.0	69	32.6	N/A		2205	51.5
Age								
Mean (SE)	68.8 (0.7)		70.9 (1.2)		75.0 (0.7)		69.3 (0.3)	
Year of survey (# of participants)								
2010	37	8.9	14	9.0	28	8.2	338	8.9
2011	34	7.6	20	11.8	34	10.6	381	9.9
2012	50	10.4	20	9.9	46	12.7	470	10.7
2013	55	13.1	14	9.3	41	12.4	454	10.6
2014	46	13.0	22	11.0	28	10.9	421	10.6
2015	41	11.0	20	10.9	33	9.5	415	11.6
2016	46	12.3	26	16.5	43	11.3	493	12.8
2017	49	12.1	20	13.3	40	11.5	471	12.6
2018	43	11.6	11	8.3	46	12.9	425	12.2
Race								
Black	127	19.6	42	16.1	126	23.8	1206	18.0
Hispanic White	69	10.0	28	8.5	40	8.3	606	8.5
Non-Hispanic White	205	70.4	97	75.4	173	68.0	2056	73.4
Education								
HS diploma/GED or less/unknown	246	57.0	116	66.1	193	50.4	2358	52.5
Some college or more	155	43.0	51	33.9	146	49.6	1510	47.4
Poverty level								
Low	177	35.0	84	41.2	122	27.8	1752	34.9
Middle	116	28.0	51	32.7	108	32.8	1154	31.6
High	108	37.1	32	26.1	109	39.4	962	33.5
Insurance coverage								
Public/uninsured	208	60.1	84	57.5	197	65.1	1836	56.1
Private	193	39.6	83	42.5	142	34.9	2032	43.9
Marital status								
Married	163	46.8	75	53.8	209	63.6	1876	54.0
Widowed	133	31.5	48	24.2	52	16.7	861	21.3
Not married	105	21.7	44	22.0	78	19.7	1131	24.6
Current smoker								
Yes	33	7.9	8	3.0	20	4.7	403	10.8
No	325	92.1	148	97.0	273	95.3	3040	89.2
Hypertension								
Yes	329	82.5	144	87.4	285	83.1	3298	83.9
No	72	17.5	23	12.6	54	16.8	570	16.1
Kidney problems								
Yes	353	87.8	144	88.9	43	83.8	485	12.5
No	48	12.2	23	11.1	296	16.2	3383	87.5
High cholesterol								
Yes	317	80.7	127	78.3	273	80.0	3029	78.9
No	84	19.3	40	21.7	66	20.0	839	21.1
CVD								
Yes	117	32.1	65	38.9	138	45.3	1419	39.8
No	284	68.0	102	61.1	201	54.7	2449	60.2

Table 2 shows weighted, unadjusted proportions, odds ratios, and 95% confidence intervals of receipt of quality-of-care indicators, which indicates significant differences by race, cancer site, and status.

### A1c testing

For A1c testing, Hispanic breast cancer survivors, were less likely to report receipt of testing (OR 0.33; 95% CI 0.15, 0.72) compared to NHW survivors.

### Foot care

For foot care, among non-cancer controls, Hispanic individuals (OR 0.79; 95% CI 0.63, 0.99) were significantly less likely to report receipt of these exams compared to NHW individuals.

### LDL testing

For LDL testing, Black (OR 0.59; 95% CI 0.48, 0.72) and Hispanic (OR 0.61; 95% CI 0.48, 0.79) non-cancer controls were significantly less likely to receive testing compared to NHWs. Across cancer survivors, Black survivors (OR 0.59; 95% CI 0.39–0.9) were less likely to receive LDL testing compared to NHW survivors. Among PCa survivors, Black survivors (OR 0.43; 95% CI 0.22, 0.84) were less likely to receive LDL tests compared to NHW survivors.

### Eye exam

In non-cancer controls, NHWs (59.1%) reported significantly (*p* = 0.004) higher rates of dilated eye exam receipt compared to Blacks (52.9%) and Hispanic (48.4%) individuals. Among PCa survivors, 73.1% of NHWs report receiving dilated eye exams compared to 61.4% of Black and 58.4% of Hispanic survivors (*p* = 0.05).

### Flu shots

Among non-cancer controls, Black (OR 0.47; 95% CI 0.38,0.58) and Hispanic (OR 0.64; 95% CI 0.5,0.81) individuals were significantly less likely to receive a flu shot compared to NHWs. Across cancer types, Black survivors (OR 0.45; 95% CI 0.3, 0.69) were less likely to receive a flu shot compared to NHW survivors. Among breast (OR 0.54; 95% CI 0.29, 1.00) and PCa (OR 0.33; 95% CI 0.18, 0.61) survivors, receipt of a flu shot was significantly less likely compared to NHW cancer survivors.

### Overall diabetes care quality

For overall care quality (receipt of four or more diabetes-related services), Black (OR 0.63; 95% CI 0.42, 0.97) and Hispanic (OR 0.58; 95% CI 0.37, 0.93) non-cancer controls were significantly less likely to reported receipt of adequate care compared to NHWs. Among PCa survivors, Black (OR 0.42; 95% CI 0.23, 0.75) and Hispanic (OR 0.43; 95% CI 0.20, 0.92) survivors were less likely to report receipt of comprehensive care compared to NHW survivors.

**Table 2:** Unadjusted weighted percentages, odds ratios and 95% confidence intervals of receipt of diabetes quality indicators by race/ethnicity and cancer site and cancer status

		2 × A1c^1^	Foot check	LDL test	Eye exam	Flu shot	Overall quality score^2^
		Weighted %	Weighted %	Weighted %	Weighted %	Weighted %	Weighted %
		OR (95% CI)	OR (95% CI)	OR (95% CI)	OR (95% CI)	OR (95% CI)	OR (95% CI)
No-cancer	Black	83.7%	54.6%	60.8%	52.9%	42.3%	43.3%
		0.93 (0.71, 1.22)	0.96 (0.89, 1.19)	**0.59 (0.48, 0.72**	0.83 (0.69, 1.01)	0.47 (0.38, 0.58)	0.90 (0.48, 1.69)
	Hispanic	83.3%	49.8%	61.9%	48.4%	50.3%	43.6%
		0.89 (0.65, 1.23)	**0.79 (0.63, 0.99)**	**0.61 (0.48, 0.79)**	**0.69 (0.54, 0.88)**	**0.64 (0.50, 0.81)**	0.58 (0.37, 0.93)
	NHW	85.6%	57.5%	73.5%	59.1%	62.4%	55.6%
		REF	REF	REF	REF	REF	REF
	***p-value***	0.30	**0.04**	** < 0.0001**	**0.0001**	** < 0.0001**	** < 0.0001**
All cancers	Black	84.4%	53.0%	64.5%	60.2%	47.8%	50.9%
		0.85 (0.5, 1.44)	0.80 (0.53, 1.20)	**0.59 (0.39, 0.90**)	1.01 (0.69, 1.51)	**0.45 (0.30, 0.69)**	0.90 (0.48, 1.69)
	Hispanic	85.5%	54.4%	69.6%	52.9%	57.4%	48.7%
		0.71 (0.41, 1.25)	0.86 (0.53, 1.41)	0.76 (0.44, 1.32)	0.84 (0.53, 1.35)	0.66 (0.38, 1.12)	0.65 (0.33, 1.26)
	NHW	87.3%	59.8%	75.9%	60.9%	68.0%	63.4%
		REF	REF	REF	REF	REF	REF
	***p-value***	0.35	0.29	**0.02**	0.42	** < 0.0001**	**0.004**
Breast	Black	91.4%	55.1%	62.6%	59.9%	48.2%	51.5%
		1.26 (0.53, 2.96)	1.38 (0.75, 2.52)	0.71 (0.40, 1.29)	1.42 (0.79, 2.53)	**0.54 (0.29, 1.00)**	0.90 (0.48, 1.69)
	Hispanic	74.7%	53.9%	65.5%	48.7%	57.5%	44.8%
		**0.33 (0.15, 0.72)**	1.30 (0.66, 2.56)	0.79 (0.37, 1.72)	0.90 (0.49, 1.65)	0.69 (0.31, 1.51)	0.65 (0.33, 1.26)
	NHW	90.7	49.7	72.0	54.2	66.8	57.5
		REF	REF	REF	REF	REF	REF
	***p-value***	**0.004**	0.70	0.28	0.48	**0.03**	0.31
CRC	Black	70.7%	40.3%	60.2%	57.6%	42.4%	44.1%
		0.50 (0.15, 1.69)	0.39 (0.13, 1.18)	0.60 (0.20, 1.78)	1.18 (0.50, 2.82)	0.50 (0.18, 1.32)	0.59 (0.20, 1.65)
	Hispanic	82.0%	61.4%	66.8%	54.1%	46.9%	50.4%
		0.85 (0.22, 3.28)	0.91 (0.26, 3.17)	0.80 (0.32, 2.00)	1.08 (0.36, 2.84)	0.68 (0.19, 2.41)	0.74 (0.27, 2.07)
	NHW	84.5%	64.7%	71.9%	54.3%	58.7%	58.8%
		REF	REF	REF	REF	REF	REF
	***p-value***	0.30	0.13	0.49	0.95	0.33	0.40
PCa	Black	82.2%	55.1%	67.8%	61.4%	49.2%	52.6%
		0.86 (0.39, 1.91)	0.55 (0.30, 1.02)	**0.43 (0.22, 0.84)**	0.59 (0.33, 1.05)	**0.33 (0.18, 0.61)**	**0.42 (0.23, 0.75)**
	Hispanic	94.1%	51.4%	77.0%	58.4%	62.9%	53.4%
		2.91 (0.80, 10.55)	0.47 (0.19, 1.20)	0.68 (0.27, 1.68)	0.51 (0.24, 1.07)	0.58 (0.27, 1.25)	**0.43 (0.20, 0.92)**
	NHW	84.7%	69.5%	83.1%	73.1%	74.7%	73.3%
		REF	REF	REF	REF	REF	REF
	***p-value***	0.28	**0.05**	**0.02**	**0.05**	** < 0.0001**	**0.0006**

^1^A1c test 2 or more times in the past year

^2^Composite score for diabetes quality reflect those who received four or more indicators (out of five)

*A1C* hemoglobin A1c testing, *OR* odds ratio, *CI* confidence interval, *OBV* office-based visit

### Annual Office-based Visits

**Fig. 1:**
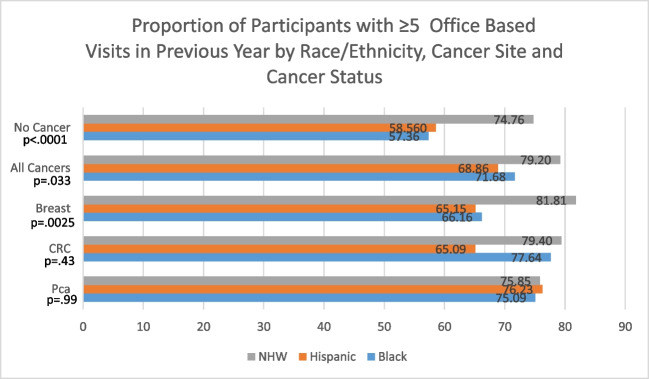
Proportion of participants with ≥ 5 office-based visits in previous year by race/ethnicity, cancer site and cancer status

Figure 1 shows the weighted percentage of respondents who had five or more office-based visits in the previous year. In the non-cancer control group, three quarters of NHWs (74.8%) reported more contact (i.e., five or more office visits in the past year) with ambulatory care providers compared Hispanic (58.5%) and Black (57.36%) individuals (*p* ≤ 0.0001). Hispanic (OR 0.5; 95% CI 0.39, 0.64) and Black (OR 0.49;95% CI 0.4, 0.6) individuals with diabetes had lower odds of having five or more office-based visits compared to NHW individuals. Among all survivors, Hispanic (OR 0.52; 95% CI 0.33, 0.83) and Black (OR 0.61; 95% CI 0.44–0.86) survivors with diabetes had significantly lower odds of having five or more office-based visits compared to NHW survivors. In breast cancer survivors, Hispanic (OR 0.47; 95% CI 0.24–0.93) and Black (OR 0.51; 95% CI 0.29, 0.9) survivors were significantly less likely to report having five or more office-based visits compared to NHW breast cancer survivors.

Final logistic regression models display weighted partially adjusted (Table 3, not adjusted for SES variables) and fully adjusted (Table 4, fully adjusted for SES and clinical variables) estimates for diabetes care quality indicators by race/ethnicity and by cancer site and status. In both the partially and fully adjusted models, interaction terms for race/ethnicity and cancer type were significant for A1c testing (*p* = 0.01); however, the interaction term for race/ethnicity by cancer status was not significant.

### Non-cancer Controls

In the partially adjusted model, we found significant race/ethnic diabetes care quality disparities for Black and Hispanic individuals compared to NHWs, both groups were less likely to receive LDL tests, flu shots or have five or more office-based visits. Black individuals were also significantly less likely to receive comprehensive diabetes care compared to NHWs. In the partially adjusted models, Black individuals were less likely to receive LDL tests (AOR 0.59; 95% CI 0.47, 0.73), flu shots (AOR 0.47; 95% CI 0.38, 0.57), comprehensive care (AOR 0.67; 95% CI 0.54, 0.83), or have five or more office-based visits (AOR 0.52; 95% CI 0.41, 0.66). In the fully adjusted models, disparities between Black and NHW individuals remained significant for each of these indices. In partially adjusted models, Hispanic patients were less likely to receive LDL testing (AOR 0.62; 95% CI 0.47, 0.81), eye exams (AOR 0.7; 95% CI 0.54, 0/9), flu shots (AOR 0.66, 95% CI 0.51, 0.86), comprehensive care (AOR 0.68, 95% CI 0.54, 0.87), and had fewer office-based visits (AOR 0.55, 95% CI 0.42, 0.72) compared to NHWs. In fully adjusted models, the disparity identified for eye exams, flu shots, and comprehensive care did not retain significance.

### All Cancers

In the partially adjusted model, we found significant racial disparities between Black and NHW survivors, with Black survivors less likely to receive LDL tests (AOR 0.56; 95% CI 0.36, 0.86), flu shots (AOR 0.44; 95% CI 0.28, 0.7), comprehensive care (AOR 0.62; 95% CI 0.4–0.96), or have five or more office-based visits (AOR 0.70; 95% CI 0.49, 0.99). Hispanic survivors were significantly less likely to receive comprehensive care (AOR 0.6; 95% CI 0.38, 0.97) compared to NHWs. In the fully adjusted models, disparities between Black and NHW cancer survivors retained significance for LDL testing and flu shot receipt.

### Breast Cancer Survivors

In the partially adjusted model, we found significant racial disparities for Black breast survivors who were less likely to receive an annual flu shot (AOR 0.49; 95% CI 0.26, 0.94) or have five or more office-based visits (AOR 0.55; 95% CI 0.3, 0.98) compared to NHW breast cancer survivors. In models fully adjusted for SES, flu shot receipt retained significance. In the partially adjusted model, Hispanic breast cancer survivors were significantly less likely to receive two A1c tests (AOR 0.24; 95% CI 0.10, 0.55) compared to NHWs. This disparity retained significance in the model controlling for SES indicators.

### Colorectal Cancer Survivors

No significant diabetes care quality disparities were identified in the CRC survivor population.

### Prostate Cancer Survivors

In the partially adjusted model, we found significant racial disparities between Black and NHW survivors for several indices: foot care, LDL tests, flu shots, comprehensive care. Black PCa survivors were less likely to receive foot checks (AOR 0.49, 95% CI 0.26, 0.95), LDL tests (AOR 0.48; 95% CI 0.24, 0.94), flu shots (AOR 0.31; 95% CI 0.16, 0.60), and comprehensive care (AOR 0.38; 95% CI 0.2, 0.72) compared to NHW PCa survivors. The disparities among Black PCa survivors retained significance after controlling for SES indices. In the partially adjusted model, Hispanic PCa survivors were significantly less likely to receive comprehensive care (AOR 0.39; 95% CI 0.17, 0.89) compared to NHW survivors. This disparity did not retain significance when controlling for SES indices.

**Table 3:** Partially adjusted logistic regression models for diabetes quality indicators by race/ethnicity and cancer status and cancer type

		2 × A1c^1,*^	Foot check	LDL test	Eye exam	Flu shot	Overall quality score^2^	OBV^3^
		OR (95% CI)	OR (95% CI)	OR (95% CI)	OR (95% CI)	OR (95% CI)	OR (95% CI)	OR (95% CI)
No cancer	Black	0.97 (0.73, 1.30)	1.00 (0.79, 1.26)	**0.59 (0.47, 0.73)**	0.82 (0.67, 1.01)	**0.47 (0.38, 0.57)**	**0.67 (0.54, 0.83)**	**0.52 (0.41, 0.66)**
	Hispanic	0.83 (0.59, 1.17)	0.83 (0.65, 1.05)	**0.62 (0.47, 0.81)**	**0.70 (0.54, 0.90)**	**0.66 (0.51, 0.86)**	**0.68 (0.54, 0.87)**	**0.55 (0.42, 0.72)**
	NHW	REF	REF	REF	REF	REF	REF	REF
All cancers	Black	0.93 (0.52, 1.66)	0.81 (0.53, 1.24)	**0.56 (0.36, 0.86)**	1.03 (0.69, 1.54)	**0.44 (0.28, 0.70)**	**0.62 (0.40, 0.96)**	**0.70 (0.49, 0.99)**
	Hispanic	0.91 (0.50, 1.66)	0.84 (0.51, 1.37)	0.91 (0.50, 1.64)	0.74 (0.48, 1.16)	0.71 (0.41, 1.24)	**0.60 (0.38, 0.97)**	0.69 (0.43, 1.12)
	NHW	REF	REF	REF	REF	REF	REF	REF
Cancer status (Y/N) vs. race	Interaction *p*-value	0.93	0.67	0.40	0.57	0.92	0.89	0.30
Breast	Black	0.97 (0.38, 2.47)	1.40 (0.73, 2.68)	0.64 (0.35, 1.17)	1.36 (0.75, 2.46)	**0.49 (0.26, 0.94)**	0.80 (0.41, 1.56)	**0.55 (0.30, 0.98)**
	Hispanic	**0.24 (0.10, 0.55)**	1.14 (0.58, 2.27)	0.81 (0.36, 1.84)	0.71 (0.41, 1.25)	0.69 (0.31, 1.53)	0.57 (0.32, 1.04)	0.60 (0.31, 1.15)
	NHW	REF	REF	REF	REF	REF	REF	REF
CRC	Black	0.58 (0.17, 2.01)	0.46 (0.15, 1.38)	0.61 (0.20, 1.90)	1.13 (0.46, 2.76)	0.50 (0.18, 1.37)	0.63 (0.21, 1.85)	0.83 (0.37, 1.86)
	Hispanic	2.08 (0.37, 11.62)	0.95 (0.32, 2.82)	1.45 (0.52, 4.00)	1.28 (0.39, 4.25)	0.73 (0.24, 2.21)	1.08 (0.35, 3.32)	0.57 (0.19, 1.67)
	NHW	REF	REF	REF	REF	REF	REF	REF
PCa	Black	1.19 (0.51, 2.78)	**0.49 (0.26, 0.95)**	**0.48 (0.24, 0.94)**	0.61 (0.33, 1.12)	**0.31 (0.16, 0.60)**	**0.38 (0.20, 0.72)**	0.94 (0.49, 1.70)
	Hispanic	3.95 (1.07, 14.64)	0.45 (0.16, 1.28)	0.84 (0.34, 2.08)	0.52 (0.24, 1.12)	0.60 (0.28, 1.31)	**0.39 (0.17, 0.89)**	0.97 (0.37, 2.59)
	NHW	REF	REF	REF	REF	REF	REF	REF
Cancer type vs. race/ethnicity	Interaction *p*-value	**0.01**	0.32	0.51	0.43	0.96	0.61	0.59

^1^A1c tested 2 or more times a year, *reference group for A1c are those who were tested < 2 times

^2^Quality score reference group = receiving 0–3 clinically indicated services

^3^OBV = 1 if 0–4 visits, 2 if 5–10 visits, 3 if > 10 visits

^4^No cancer was the reference for both models (cancer status and cancer type)

^5^Cancer type = breast/CRC/PCA/non-cancer

*A1C* hemoglobin A1C testing, *AOR* adjusted odds ratio, *CI* confidence interval, *OBV* office-based visit

Models were adjusted for age, sex, smoking status, and history of hypertension, kidney disease, high cholesterol and CVD; Note, socioeconomic status variables (poverty status, education, marital status, insurance status) were not adjusted in this model

**Table 4:** Fully adjusted logistic regression models for diabetes quality indicators by race/ethnicity and cancer status and cancer type

		2 × A1c^1,*^	Foot check	LDL test	Eye exam	Flu shot	Overall quality score^2^	OBV^3^
		OR (95% CI)	OR (95% CI)	OR (95% CI)	OR (95% CI)	OR (95% CI)	OR (95% CI)	OR (95% CI)
No cancer^4^	Black	1.01 (0.75, 1.36)	1.01 (0.79, 1.27)	**0.63 (0.51, 0.78)**	0.97 (0.79, 1.19)	**0.52 (0.42, 0.64)**	**0.73 (0.59, 0.90)**	**0.59 (0.46, 0.75)**
	Hispanic	0.85 (0.60, 1.20)	0.87 (0.67, 1.12)	**0.76 (0.58, 1.00)**	0.93 (0.71, 1.22)	0.84 (0.63, 1.11)	0.82 (0.63, 1.07)	**0.70 (0.52, 0.94)**
	NHW	REF	REF	REF	REF	REF	REF	REF
All cancers	Black	0.96 (0.54, 1.72)	0.82 (0.53, 1.25)	**0.58 (0.38, 0.89)**	1.16 (0.79, 1.71)	**0.47 (0.31, 0.74)**	0.66 (0.43, 1.03)	0.77 (0.54, 1.09)
	Hispanic	0.95 (0.52, 1.71)	0.86 (0.52, 1.40)	1.00 (0.56, 1.80)	0.87 (0.55, 1.39)	0.81 (0.47, 1.42)	0.68 (0.41, 1.10)	0.79 (0.50, 1.24)
	NHW	REF	REF	REF	REF	REF	REF	REF
Cancer status (Y/N) vs. race	Interaction *p*-value	0.92	0.66	0.51	0.57	0.95	0.75	0.46
Breast	Black	0.99 (0.39, 2.53)	1.39 (0.72, 2.66)	0.63 (0.34, 1.14)	1.48 (0.84, 2.63)	**0.51 (0.27, 0.96)**	0.84 (0.44, 1.60)	0.58 (0.32, 1.05)
	Hispanic	**0.25 (0.11, 0.58)**	1.15 (0.57, 2.26)	0.85 (0.37, 1.95)	0.80 (0.45, 1.44)	0.76 (0.34, 1.72)	0.62 (0.34, 1.16)	0.67 (0.36, 1.25)
	NHW	REF	REF	REF	REF	REF	REF	REF
CRC	Black	0.58 (0.17, 2.03)	0.46 (0.15, 1.40)	0.58 (0.19, 1.80)	1.13 (0.46, 2.82)	0.48 (0.18, 1.29)	0.62 (0.21, 1.87)	0.82 (0.37, 1.82)
	Hispanic	2.10 (0.34, 12.98)	0.95 (0.32, 2.79)	1.33 (0.46, 3.81)	1.40 (0.40, 4.89)	0.78 (0.26, 2.35)	1.13 (0.35, 3.65)	0.59 (0.21, 1.70)
	NHW	REF	REF	REF	REF	REF	REF	REF
PCa	Black	1.22 (0.54, 2.78)	**0.50 (0.26, 0.97)**	**0.45 (0.23, 0.89)**	0.75 (0.41, 1.36)	**0.36 (0.19, 0.69)**	**0.43 (0.23, 0.80)**	1.09 (0.58, 2.05)
	Hispanic	3.81 (1.04, 13.86)	0.48 (0.17, 1.36)	0.83 (0.33, 2.08)	0.66 (0.28, 1.51)	0.73 (0.33, 1.60)	0.45 (0.19, 1.06)	1.21 (0.49, 2.98)
	NHW	REF	REF	REF	REF	REF	REF	REF
Cancer type^4^ vs. race/ethnicity	Interaction *p*-value	**0.01**	0.36	0.78	0.63	0.99	0.64	0.59

^1^A1c tested 2 or more times a year, *reference group for A1c are those who were tested < 2 times

^2^Quality score reference group = receiving 0–3 clinically indicated services

^3^OBV = 1 if 0–4 visits, 2 if 5–10 visits, 3 if > 10 visits

^4^No cancer was the reference for both models (cancer status and cancer type)

^5^Cancer type = breast/CRC/PCA/Non-cancer

*A1C* hemoglobin A1C testing, *AOR* adjusted odds ratio, *CI* confidence interval, *OBV* office-based visit

Models were adjusted for age, sex, poverty status, education, marital status, insurance status, smoking status, and history of hypertension, kidney disease, high cholesterol and CVD

## Discussion

In this national sample of individuals with diabetes, DCQ disparities among age, race, and data collection year matched non-cancer controls were pronounced and evident among cancer survivor populations in 2010–2018. In models that did not adjust for SES variables, the odds of receipt of care were lower for Black and Hispanic non-cancer controls for several specific indices (LDL tests, flu shots, eye exams (Hispanics only)), overall DCQ compared to NHWs. Among all cancer survivors’ groups, disparities for Blacks were identified for specific diabetes care indices, overall care quality, and office-based visits when SES was not controlled. Black breast cancer survivors were less likely to receive flu shots or have five or more office-based visits compared to NHWs. In a previous study, Black women with breast cancer and diabetes had low rates of eye and foot care and suboptimal A1c management [29]. Our findings did not indicate racial disparities for these specific indices, but found flu shot receipt disparities between Black and White breast cancer survivors. Hispanic breast cancer survivors were less likely to receive A1c testing compared to NHW breast cancer survivors, consistent with a disparity found among the general population [30]. Among PCa survivors, both Black and Hispanic survivors were less likely to report receipt of comprehensive care compared to NHW survivors. Risk-based care delivery models have been proposed as a strategy to align comprehensive care needs with care receipt among cancer survivors; however, in the US, these models have not been implemented [31]. While these are often described in the context of post-cancer diagnosis care our findings suggest that aligning risk with care delivery should begin prior to cancer diagnosis.

These findings highlight the need for responsive strategies to address intersectional racial, ethnic, and SES barriers to care that structurally disadvantage minoritized populations that disproportionately manage multiple chronic conditions [32]. The present study adds observable differences inequitable diabetes care delivery for cancer survivors and non-cancer controls by socioeconomic status, race, and ethnicity which can inform future research about why these cumulative disparities exist and how to address these inequities through policies and program development. Nearly three-quarters of NHW non-cancer controls had five or more office-based visits in the previous year, a proportion greater than both Hispanic (68.9%) and Black (71.2%) cancer survivors with diabetes. Therefore, Black and Hispanic cancer survivors, who are potentially managing cardiometabolic multi-morbidity have fewer office visits (and presumably less care) than non-cancer control NHWs managing diabetes alone. Infrequent office visits (less than eight per year) are associated with suboptimal diabetes care quality among patients with diabetes [33]. Guiding principles to advance equitable care delivery research emphasize the need to (1) recognize racism as a fundamental driver of healthcare inequities, (2) engage community members and other relevant partners, (3) include multisector partnerships, and (4) recognize the centrality of context [34]. Recognizing racism as a fundamental driver of the inequities identified for both cancer survivors and non-cancer controls frames race and ethnicity through a social contextual lens [32]. This framing encourages the interrogation of the health policy, social policy, and healthcare institutional decisions that presently and historically perpetuate these care disparities. In the US, diabetes and cancer care are expensive to manage independently of one another—financial costs and illness-related hardship impacts cancer screening rates, diabetes self-management behaviors, access to and consistent use of diabetes medications, access to primary care for chronic disease prevention and management, and timely access to cancer care [35–37]. Access to care and SES are interrelated, greater disease burden for both cancer and diabetes is associated with low SES, and both require resources to effectively prevent and manage [38]. Macro-level social and health policy decisions have implications for the availability of the resources to effectively prevent and manage identified disparities through the allocation and redistribution of resources. Policy decisions can incentivize the use of care delivery strategies that address social risks, remove barriers to care access, and improve SES among patients with diabetes (with and without cancer) have the potential to reduce diabetes disparities among populations managing diabetes [39].

In our accounting of care delivery context, we rely on insights from previous research which has described the most common care team composition for early-stage breast cancer survivors with cardiopulmonary and diabetes comorbidities as involving primary care and oncology [40]. This study also identified access to medical sub-specialty care as a possible mechanism for racial/ethnic disparities among survivors with comorbidities, with lower rates of high care complexity (which includes more specialists) among African American and dual recipients of Medicare–Medicaid (compared to non-Hispanic Whites and Medicare-only, respectively) [40]. Greater understandings of the impact of the care team capacities and functioning across primary care and oncology would provide insights into whether these teams prioritize addressing social needs known to contribute to access to care and other delivery disparities [41]. Care disparities among non-cancer controls are consistent with known racial/ethnic disparities in diabetes care quality [18]. Cancer care teams struggle to respond to diabetes care needs and opportunities to assess and intervene at the point of diagnosis and initiation of cancer treatment are routinely missed [42]. Racial and ethnic disparities among the non-cancer controls suggest that the cancer care delivery deficit to be responsive to diabetes management may disproportionately impact health disparities populations—compounding care disparities as care complexity increases with a diagnosis of cancer.

In 2020, the National Cancer Institute posed the provocative question: “What strategies improve and sustain coordination of comprehensive healthcare for underserved cancer patients with comorbid conditions?” To date, there has been scant research using epidemiological data that provides insight into potential change levers to promote health equity for cancer populations co-managing diabetes. These studies are often limited in understanding the disease trajectory—for either cancer or diabetes—or restricted to a specific age group. A population-based US study of 67 + year-old cancer survivors (*n* = 34,643) identified racial/ethnic disparities in diabetes care processes of care among breast, colorectal, and PCa survivors in the 12 months after an incident cancer diagnosis [43]. Using SEER data linked to Medicare claims this study found that compared to NHWs, Black cancer survivors were less likely to receive A1c test, LDL tests, and annual eye exams [43]. In that nationally representative sample, Blacks and Hispanics were less likely to receive all three recommended annual tests compared to NHWs and these disparities persisted across breast, colorectal, and prostate cancer types [43]. The present study found similar care disparities for specific indices, suggesting that care delivery research to align supportive care strategies with cumulative medical and social risks is a necessary next step. Researchers have found high rates of fragmented care (78.5%) among Black breast cancer survivors with comorbidities [44]. Specifically, majority of the African American breast cancer survivors received primary care and oncology care from different health systems, which can lead to disconnects in care [44]. Strategies to improve coordination between primary care and oncology for general survivor populations—care plans and web-based tools—have not yielded improvements in care delivery or health outcome [45, 46]. Strategies that align care coordination and resources should be tailored based on patient needs and cumulative risks. Care coordination and patient navigation was endorsed by an expert roundtable as an actionable approach to address health equities in care delivery [47].

Notably, the estimates of care disparities from the current study are lower than those reported by larger studies of cancer survivors [43]. These higher rates may reflect the advanced age, larger sample sizes, and the proximity to initial diagnosis (one year) on which most studies focus [43, 44]. These studies offer insights about the initial cancer treatment phase of care which is particularly stressful, when patients are managing the uncertainty of their cancer treatment outcome, financial burden, and physical toll of treatment. Our study adds to the broader understanding of how diabetes care disparities also affect longer term cancer survivor population and may have a cumulative impact for underserved populations [44]. Additionally, given the profound disparities in the non-cancer control population, it may be more impactful to address diabetes care disparities prior to cancer diagnosis. One study found that among a cohort of Black breast cancer survivors, optimal A1c < 8.0% was achieved for less than 50% of the patients prior to diagnosis and in the year post-diagnosis [44]. Another study found that among non-metastatic breast, prostate, or colorectal cancer survivors with diabetes, Black individuals with cancer and diabetes were more likely to have an emergency department visit and to be hospitalized between 1 and 2 years post-diagnosis compared to NHWs [48]. Our findings are representative of longer term survivors’ experiences and provides insights into where focused attention is needed for these survivor populations.

Based on our findings, Black prostate cancer survivors with diabetes may be at greater risk for disparities in DCQ over the longer term phases of survivorship. Black men have similar age-adjusted prevalence for diabetes compared to Black women, but greater rates overall are compared to NHW individuals [11, 49]. Nationally, rates of diabetes-related complications are decreasing overall; however, these continue to rise among Black and Hispanic populations [50, 51]. Black men represent an understudied population within both diabetes and prostate cancer. A recent synthesis of the state of the evidence concluded that there is a weak foundational knowledge on which factors facilitate or hinder diabetes self-management among Black men [52]. A recent review reported that non-Hispanic Black men only represented an average of 15% of sample sizes on diabetes self-management research focused on Black populations over the past 20 years [53]. Lack of trust in research and access barriers are among the cited reasons Black men are underrepresented in prostate cancer research [54]. Innovative strategies to promote recruitment of Black men into health promotion programming and clinical research (i.e., recruiting in trusted environment, tapping into existing social networks, appealing to join a broader movement for social change) provide a path forward to rectify knowledge gaps in both prostate cancer survivorship and diabetes management [55–57].

Though this study has several strengths, including the examination of diabetes care quality disparities by cancer type, there are several notable limitations. First, we used retrospective data where self-reports of race and ethnicity recognizing that the concept or “race” and “ethnicity” are conceptually complex and inter-related with other confounders. We recognize that excluding other racially and ethnic groups that had limited sample sizes limits the studies scope [58]. Second, we include patients with type I and II diabetes in our analysis. The evidence of increased CRC risk among patients with type I diabetes is not conclusive and there have been critiques about the generalizability of CRC cancer risk from the broader population of patients with type II diabetes compared to those with type I [59]. Third, data used to assess co-morbidity and other covariates were self-reported and therefore potentially subject to recall and social desirability bias. The MEPS data set does not have data available about the timing of the diabetes and cancer diagnosis which limits analyses about the temporal relationship between these diagnoses on poorer outcomes. This is important as diabetes during the acute cancer treatment phase may influence receipt of guideline concordant cancer treatment which can impact overall outcomes [55]. Fourth, we used statistical matching analyses to improve the comparability between those who have had a cancer diagnosis and the broader context of diabetes and multi-morbidity in the wider population [60]. While we attempted to reach a 5:1 match, we could not obtain a full five non-cancer cases for each cancer case because of the limited number of individuals with diabetes of each race in the specific years in the data set. Fifth, the MEPS data set does not collect cancer stage data. Sixth, this data was collected pre-pandemic—a period where systems were tested and care was disrupted and minority and low socioeconomic patients were more likely to experience missed or delayed care. Seventh, these findings are representative to the US population but may not be generalizable to other health systems or countries. Despite these limitations, this study illuminates the complexities of diabetes care disparities that may impact cancer survivorship for groups underrepresented in currently implemented cancer survivorship models—including low socioeconomic status and racial/ethnic minorities groups. Specifically, this study highlights the need for further attention to investigate disparities in multi-morbidity among aging racial/ethnic minority populations during the prevention phase of cancer care delivery.

In conclusion, the disparities identified among cancer survivors have implications for primary care and oncologic care delivery. Care models promoting health equity to prevent and manage complex cardiometabolic’ clusters of multi-morbidity that address racial, ethnic and socioeconomic diabetes care delivery disparities are needed [61]. There remains a large gap in research related to complex cardiometabolic cancer survivors and their care coordination needs. This past decade, healthcare systems have consolidated with the goal of producing better care coordination and care quality; however, the impact remains mixed. Strong care delivery models that can address the pandemic impact in the long term and can be prepared for possible future disruptions are needed. More research is needed to understand the implications of these system changes for complex cardiometabolic cancer survivors who are disproportionate low SES and racial and ethnic minorities [62–64].

## Data Availability

This data is publicly available through the free IPUMS health surveys service found at IPUMS Health Surveys.

## Code Availability

Data was analyzed using SAS (SAS Enterprise Guide 7.1). Please contact the corresponding author for SAS code utilized.
